# Receptor architecture of the macaque lateral geniculate nucleus

**DOI:** 10.1007/s00429-026-03109-5

**Published:** 2026-05-19

**Authors:** Marina Saito, Lucija Rapan, Meiqi Niu, Ling Zhao, Sei-ichi Tsujimura, Hiromasa Takemura, Nicola Palomero-Gallagher

**Affiliations:** 1 Faculty of Design and Architecture, Nagoya City University, 2-1-10, Kita Chikusa, Chikusa-ku, Nagoya, 464−0083 Japan; 2 Division and Sensory and Cognitive Brain Mapping, Department of System Neuroscience, National Institute for Physiological Sciences, Okazaki, 444–8585 Japan; 3 Japan Society for the Promotion of Science, Tokyo, 102−0083 Japan; 4 Laboratory for Imagination and Executive Functions, RIKEN Center for Brain Science, Wako, 351−0198 Japan; 5 C. & O. Vogt Institute of Brain Research, Heinrich-Heine University Düsseldorf, 40225 Düsseldorf, Germany; 6 Research Centre Jülich, Institute of Neuroscience and Medicine (INM-1), 52425 Jülich, Germany; 7 Department of Psychology, School of Public Policy and Management, Nanchang University, Nanchang, 330031 China; 8 The Graduate Institute of Advanced Studies, SOKENDAI, Hayama, 240−0115 Japan; 9 Core for Spin Life Sciences, Okazaki Collaborative Platform, National Institutes of Natural Sciences, Okazaki, 444–8585 Japan

**Keywords:** Lateral geniculate nucleus, Receptor autoradiography, Primary visual cortex, Neurotransmitter receptor, Acetylcholine, Visual system

## Abstract

The lateral geniculate nucleus (LGN) is a key thalamic nucleus in the primate visual system that relays visual information from the retina to cortical visual areas. While functional and microanatomical characteristics of the LGN and its layers have been extensively studied, previous investigations on its receptor architecture have been restricted to a small subset of neurotransmitter receptors. To characterise the receptor architecture of the macaque LGN in greater detail, we analysed in vitro autoradiography data to quantify the density of 15 neurotransmitter receptors at the sublayer level, thus improving our understanding of the molecular architecture supporting primate visual function. For comparison, we also determined the densities of these receptors in the primary visual cortex (V1) at a laminar level. Though comparable in shape, the receptor fingeprints of magnocellular layers were larger than those of parvocellular layers. In contrast, receptor fingerprints of cytoarchitectonic layers in V1 differed in both shape and size. Ionotropic/metabotropic and excitatory/inhibitory receptor ratios were significantly larger in V1 than in the LGN, suggesting a greater prominence of inhibitory neurotransmission in the latter region. The nicotinic α_4_β_2_ receptor was the only receptor type with a higher density in the LGN compared to V1, highlighting the particular importance of acetylcholine in the modulation of visual stimuli. These findings provide insights into the receptor architecture underlying functions of the LGN, with implications for mechanisms such as visual signal encoding and surround suppression.

**Supplementary Information:**

The online version contains supplementary material available at 10.1007/s00429-026-03109-5.

## Introduction

Vision is a fundamental sensory modality for humans and other primate species, enabling the integration of information from the environment, avoidance of danger, and communication with other individuals (Leopold et al. 2020). Accurate knowledge concerning the relationship between the visual system’s function and anatomical organisation constitutes an unavoidable prerequisite for understanding biological mechanisms on how primates, including humans, obtain information about the external world and how damage to specific components of the visual system leads to dysfunction in visual function.

The lateral geniculate nucleus (LGN) is a thalamic nucleus known to play a key role in relaying signals from retinal ganglion cells to the visual cortex in the primate visual system (Nassi and Callaway 2009). Previous neuroanatomical and electrophysiological studies have investigated the LGN in macaque monkeys because of the close similarity of their visual system to that of humans (De Valois and Jacobs 1968). These studies identified that the LGN is composed of three types of sublayers (magnocellular, parvocellular, and koniocellular), each with distinct functional and anatomical properties (Derrington and Lennie 1984). Based on their functional properties, vision scientists have proposed that different LGN sublayers play distinct roles in visual processing. For instance, researchers proposed hypotheses regarding the magnocellular contribution to motion perception (Livingstone and Hubel 1988) and reading (Demb et al. 1998; Main et al. 2014; Livingstone et al. 1991). Because of its critical role in visual processing, the LGN has been a frequent subject of neuroimaging studies aiming to shed light on visual function (Denison et al. 2014; Muller-Axt et al. 2021; Oishi et al. 2023) as well as of studies evaluating the impact of retinal or cortical lesions on visual processing (Joffe et al. 1997; Gupta et al. 2009; Schmid et al. 2010; Bridge et al. 2019).

The anatomical connectivity of the LGN is an important feature that conditions its role in visual processing, and has also been extensively studied. Previous studies found that the axons of LGN projection neurons mainly target layers IVCα and IVCβ of the primary visual cortex (V1), although they have collaterals terminating in other layers (Hendrickson et al. 1978; Fitzpatrick et al. 1983; Hendry and Reid 2000). The feedback connectivity from V1 to the LGN stems from the so-called corticogeniculate neurons of layer VI (Briggs et al. 2016; Fitzpatrick et al. 1994). In contrast to this rich knowledge concerning LGN connectivity and functions, a detailed characterisation of the molecular basis supporting visual functions mediated by the LGN and its sublayers remains to be seen.

Neurotransmitters and their receptors constitute key elements in signal transduction in the brain and thus provide the molecular infrastructure to understand functional neuroanatomy (Zilles et al. 2004). The heterogeneous distribution of receptors for classical neurotransmitters, both at the regional and laminar level, serves to delineate cortical areas and subcortical nuclei, segregates cortical types and functional systems, and highlights hierarchical processing levels within a given functional system (Palomero-Gallagher and Zilles 2019; Zilles and Palomero-Gallagher 2017b). Importantly for translational neuroscience, these organisation principles not only apply to the human brain but are evolutionarily conserved (Zilles and Palomero-Gallagher 2017a). The architecture of neurotransmitter receptors in macaque LGN has been investigated using immunochemistry (Cimino et al. 1992; Jones et al. 1998) or autoradiography (Shaw and Cynader 1986; Cimino et al. 1992; Ibrahim et al. 2000; Perez-Santos et al. 2021). In addition, few studies also investigated receptor architecture in human LGN (Oke et al. 1997; Spurden et al. 1997; Hurd et al. 2001; Waldvogel et al. 2017). However, because each of these studies examined only a small subset of neurotransmitter receptors, an accurate understanding of the receptor architecture in the primate LGN requires an analysis that more comprehensively covers multiple receptor types and neurotransmitter systems.

In this study, we aimed to systematically characterise the receptor architecture of the macaque LGN with 15 receptor types for 6 neurotransmitters by means of in vitro receptor autoradiography to better understand the substrate of early visual functions in the primate brain. Previous studies have shown that cortical areas within the same functional system tend to share similar receptor fingerprints, while those from different systems diverge (Zilles et al. 2015; Palomero-Gallagher and Zilles 2018). Whether this principle extends into the subcortical components of such networks remains unknown. Here, we present the first systematic test of receptor fingerprint similarity between cortical and subcortical components of a functional network. The LGN and V1 provide an ideal starting point for such an analysis, as their laminar patterns of connectivity are well established (Briggs 2020) and offer a clear framework for evaluating cross-level correspondences.

## Materials and methods

### Dataset

We analysed data obtained from three adult male macaque monkey brains (*Macaca fascicularis*; brains ID: 11530, 11539, 11543; 6 ± 1 years of age; obtained from Covance Laboratories, Münster, Germany). All the protocols in this study, which did not include any experimental procedures with living animals, had the approval of the Institutional Animal Care and Use Committee, were carried out in accordance with the European and local Committees, and complied with the European Communities Council Directive 2010/63/EU.

The brains were divided into left and right hemispheres, which were further separated into an anterior and a posterior slab at the height of the most caudal part of the central sulcus. All slabs were shock frozen in N-methylbutane (isopentane) at -40 °C for 10–15 min, and serially sectioned (thickness 20 μm) in the coronal plane with a cryotome at  -20 °C. Sections were thaw-mounted on gelatin-coated glass slides, air dried and stored at -80 °C for further processing.

**Fig. 1:**
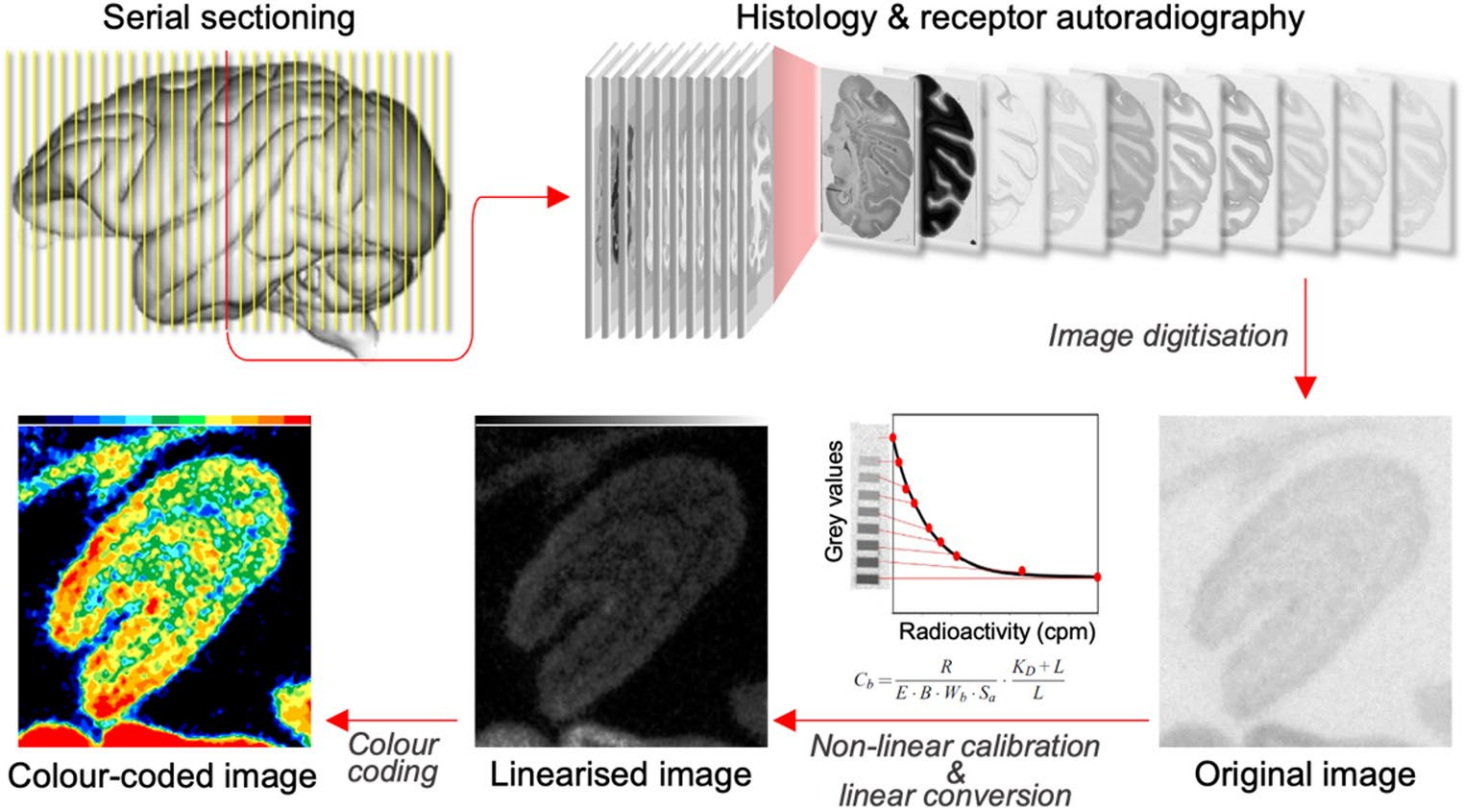
**Workflow for the generation of autoradiographs for the visualisation and quantification of receptor densities in the lateral geniculate nucleus**. Histological processing and receptor autoradiography were performed on brain sections from macaque monkeys. After digitisation of the receptor autoradiographs ensuing from the binding experiments, grey values in each image were linearised to obtain quantitative measures of receptor density, expressed in fmol/mg protein. For visualisation purposes, the linearised image was colour-coded without altering the quantitative scale. Abbreviations: Cb: concentration of binding sites; R: amount of radioactivity; E: efficiency of the scintillation counter; B: number of decays per unit of time and radioactivity; Wb: protein weight of a standard; Sa: specific activity of the radiolabelled ligand; KD: dissociation constant of the ligand; L: free concentration of the radiolabelled ligand during incubation

Sections were processed to visualise the laminar and regional distribution patterns of cell bodies using classical silver cell-body histological staining (Merker 1983), as well as the distribution and density of 15 receptor types belonging to the classical neurotransmitters glutamate, GABA, acetylcholine, noradrenaline, serotonin and dopamine by means of quantitative in vitro receptor autoradiography (Palomero-Gallagher and Zilles 2018; Zilles et al. 2002b). Sections were arranged into sequential series, each consisting of 24 sections (the 16 modalities analysed here plus 8 reserve sections; Fig. 1), and separated from each other by approximately 400 μm. This sequence was repeated throughout the entire rostro-caudal extent of the hemisphere. Given that the LGN spans approximately 6 mm in its rostro-caudal extent (it is located between Bregma levels -12.15 mm and -18 mm according to Paxinos et al. (2023) and between Bregma levels + 11 mm and + 5 mm according to Saleem and Logothetis (2012), this sampling strategy yielded 3–5 sections per modality (cell body stain or one of the 15 analysed receptor types) and hemisphere, which were used for the final analysis. Receptor autoradiography was performed according to previously published protocols (Palomero-Gallagher and Zilles 2018; Zilles et al. 2002b). In brief, the protocol encompassed a preincubation for section re-hydration and removal of endogenous substances, a main incubation for receptor labelling by means of a subtype-specific tritiated ligand (Table 1), and a rinsing step to stop the binding process. The radiolabelled sections were then air dried and exposed against tritium-sensitive films (Hyperfilm, Amersham, Braunschweig, Germany) together with plastic tritium standards of known radioactivity concentrations (Microscales^®^, Amersham) for 4–18 weeks. The ensuing autoradiographs reveal the regional and laminar distribution of receptor binding sites.

**Table 1:** List of the analysed receptor types, the tritiated ligand used to label them, the effect that their activation has on the membrane potential, and their mechanism of action

Neurotransmitter	Receptor	Effect	Mechanism	[^3^H] ligand
Glutamate	AMPA	excitatory	ionotropic	AMPA
	kainate	excitatory	ionotropic	kainate
	NMDA	excitatory	ionotropic	MK-801
GABA	GABA_A_	inhibitory	ionotropic	muscimol
	GABA_A_/BZ	inhibitory	ionotropic	flumazenil
	GABA_B_	inhibitory	metabotropic	SR95531
Acetylcholine	muscarinic M_1_	excitatory	metabotropic	pirenzepine
	muscarinic M_2_	inhibitory	metabotropic	oxotremorine-M
	muscarinic M_3_	excitatory	metabotropic	4-DAMP
	nicotinic α_4_β_2_	excitatory	ionotropic	epibatidine
Noradrenaline	α_1_	excitatory	metabotropic	prazosin
	α_2_	inhibitory	metabotropic	UK 14,304
Serotonin	5-HT_1A_	inhibitory	metabotropic	8-OH-DPAT
	5-HT_2_	excitatory	metabotropic	ketanserin
Dopamine	D_1_	excitatory	metabotropic	SCH23390

Histologically processed sections were scanned by means of a light microscope with a motor-operated stage and a CCD camera connected to the image acquisition and processing system KS400 (Zeiss). The in-plane spatial resolution of the resulting 8-bit images was 1 μm per pixel, thus enabling cytoarchitectonic identification of LGN and V1 layers.

Autoradiographs were digitised with an image analysis system consisting of a source of homogenous light and a CCD camera with an S-Orthoplanar 60-mm macro lens corrected for geometric distortions, connected to the image acquisition and processing system Axiovision, to enable densitometric analysis of binding site concentrations in the receptor autoradiographs (Palomero-Gallagher and Zilles 2018; Zilles et al. 2002a). The spatial resolution of the resulting images was 3000 × 4000 pixels (8-bit grey value resolution). These images only code grey values, not receptor densities. Thus, regression curves were generated that incorporated the experimental parameters for each receptor type (including the specific activity and concentration of the radiolabelled ligand, as well as its dissociation constant) together with the radioactivity concentrations of the co-exposed standards and their corresponding grey values (Fig. 1). These calibration curves were then used to linearise the grey value of each pixel in an autoradiograph and convert it into a quantitative measure of receptor density, expressed as fmol/mg protein (Palomero-Gallagher and Zilles 2018; Zilles et al. 2002b). Finally, in order to provide a clear visualisation of the regional and laminar receptor distribution patterns, digitised autoradiographs were linearly contrast-enhanced and pseudo-colour-coded (Fig. 1).

### Identification of regions-of-interest and quantification of receptor densities

**Fig. 2:**
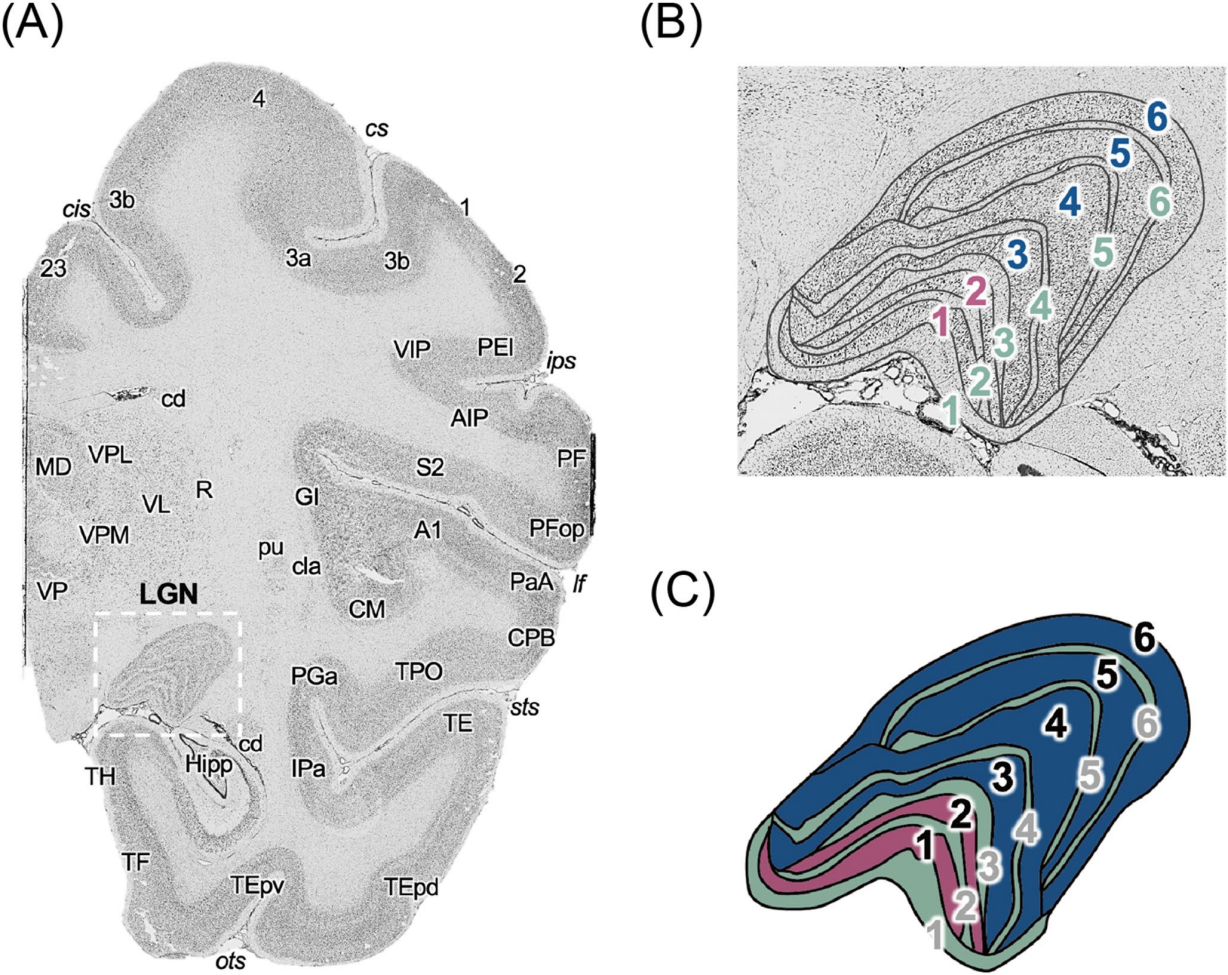
**Silver cell-body stained image of an exemplary coronal section encompassing the macaque LGN**. (**A**) Representative silver cell-body stained coronal section (section #29; brain #rh11530). The rectangle indicates the portion of the section including the LGN, which is magnified in the right panel. The anatomical labels on brain regions and sulci were defined based on the atlas by Saleem and Logothetis (2012) and parcellations of the MEBRAINS atlas (Niu et al. 2020, 2021; Rapan et al. 2021). (**B**) Magnified image of the macaque LGN. Black contours depict the manually delineated borders between sublayers (magnocellular, parvocellular, and koniocellular). (**C**) Schematic illustration of the three types of sublayers of the LGN (magenta, magnocellular; blue, parvocellular; green, koniocellular). Black and light grey numbers correspond to magnocellular/parvocellular and koniocellular layers, respectively. 1: somatosensory area 1, 2: somatosensory area 2, 23: area 23, 3a: somatosensory area 3a, 3b: somatosensory area 3b, 4: primary motor cortex, A1: primary auditory cortex, AIP: anterior intraparietal area, cd: caudate, cis: cingulate sulcus, cla: claustrum, CM: caudomedial, belt region of the auditory cortex, CPB: area CPB, cs: central sulcus, GI: granular insula, Hipp: Hippocampus, IPa: area IPa, ips: intraparietal sulcus, lf: lateral fissure, MD: mediodorsal nucleus, ots: occipital temporal sulcus, PaA: area PaA, PEI: area PEI, PF: area PF, PFop: area PFop, PGa: area PGa, pu: putamen, R: reticular nucleus, S2: secondary somatosensory area, sts: superior temporal sulcus, TE: area TE, TEpd: doral subregion of posterior TE, TEpv: ventral subregion of posterior TE, TF: area TF of the parahippocampal cortex, TH: area TH of the parahippocampal cortex, TPO: area TPO, VL: ventral lateral nucleus, VIP: ventral intraparietal area, VP: ventral pallidum, VPL: ventral posterior lateral nucleus, VPM: ventral posterior medial nucleus

We first identified the position of the entire LGN in the images of the cell-body stained sections (Fig. 2A, highlighted in the dashed rectangle) based on knowledge of its anatomical position (Paxinos et al. 2023; Saleem and Logothetis 2012), then manually delineated the border between the magnocellular, parvocellular, and koniocellular LGN sublayers (Fig. 2B and C). We used these manually delineated sublayers as regions of interest (ROIs) for subsequent analyses of receptor densities, by aligning the cytoarchitectonically-defined ROI to the receptor autoradiographic images of the neighbouring sections. The mean of the grey values contained within each ROI was extracted and transformed into a receptor concentration per unit protein (fmol/mg protein) using the in-house software AnaRec (Impieri et al. 2019). In addition to the main analyses, we also performed a supplementary analysis to compare the medial and lateral parts of the LGN (Figure S1A). To avoid reduced sensitivity due to smaller ROI sizes, for these additional analyses we did not use layer-specific ROIs. Instead, we defined an ROI including the magnocellular and adjacent koniocellular layers (the magnocellular compartment) as well as an ROI including the parvocellular and adjacent koniocellular layers (the parvocellular compartment), and subdivided each one into medial and lateral portions. The four ensuing ROIs were called MM, LM (medial and lateral parts of the magnocellular compartment, respectively), MP, and LP (medial and lateral parts of the parvocellular compartment, respectively).

To extract receptor densities from the cytoarchitectonic layers of V1 (Rapan et al. 2022), we first manually defined the outer (following the pial surface) and inner (at the border between layer VI and the white matter) contours of V1 on the receptor autoradiographs and used these lines to define equidistant traverses running perpendicularly to the cortical surface. The laminar changes in grey values were extracted along these traverses in the form of receptor profiles, which were then divided into discrete sectors corresponding to cytoarchitectonic layers by comparison with the neighbouring cell-body stained sections (Palomero-Gallagher and Zilles 2018). Receptor densities of V1 layers were obtained by computing the surface below each of these sectors using in-house developed MATLAB (The MathWorks, Inc., Natick, MA) scripts.

Mean densities were obtained for each layer through the analysis of the entire rostro-caudal extent of the LGN and of a series of 4–5 equidistantly spaced sections through V1 of each animal and receptor type, and represented as a receptor fingerprint (Zilles et al. 2002a). We generated two different types of receptor fingerprints; one visualises raw receptor density data (fmol/mg protein) and the other shows the log-scaled data (log^10^(fmol/mg protein)).

### Experimental design and statistical analyses

#### Comparison of the receptor distribution within LGN layers and between LGN and V1

Stepwise linear mixed-effects models were performed on the LGN receptor density data using R (version: 4.0.4). The model consisted of fixed effects for the LGN layer and receptor type, and the hemisphere was set as a random effect. The following Eq. (1) was used for analysis:

1 $$\:{D}_{l,r,h}=\:{\alpha\:}_{0}+\:{\alpha\:}_{1}{L}_{l}+\:{\alpha\:}_{2}{R}_{r}+\:{\alpha\:}_{3}{L}_{l}{R}_{r}+\:{\beta\:}_{1}{H}_{h}\:\:\:$$

where *D* is the receptor density, *L* is layer type (magnocellular, parvocellular, and koniocellular), *R* is receptor type, and *H* is hemisphere.

After performing the analysis of this model, the statistical results of an omnibus test showed that the interaction effect between layer and receptor type did not reach statistical significance, while the main effect of layer type showed a significant effect. Therefore, we performed a post hoc analysis to determine the significant differences in receptor densities between sublayers of LGN. The false discovery rate (FDR) approach (Benjamini and Hochberg 1995) was used for multiple comparisons in post hoc analyses.

To compare the different receptor distributions between LGN and V1, the mean receptor densities of the entire LGN and V1 were first calculated. Then, the normalised data was used for the analysis of a newly constructed model (Eq. 2), where the area (LGN and V1) and receptor type were set as fixed effects, and the hemisphere as a random effect:

2 $$\:{D}_{l,r,h}=\:{\alpha\:}_{0}+\:{\alpha\:}_{1}{A}_{a}+\:{\alpha\:}_{2}{R}_{r}+\:{\alpha\:}_{3}{A}_{a}{R}_{r}+\:{\beta\:}_{1}{H}_{h}$$

where *D* is the receptor density, *A* is the area, *R* is the receptor type, and *H* is the hemisphere.

As the interaction effect between region and receptor type was significantly different, subsequently, the simple effect test was conducted to determine whether there was a significant difference in receptor density between LGN and V1 for each receptor type. The FDR correction was also used in this simple effect analysis for multiple comparisons.

#### Comparison of the receptor distribution within medial and lateral sides of the LGN

To examine whether receptor distributions differ between the medial and lateral parts of the LGN, which represent distinct portions of the visual field, we performed a supplementary analysis to determine the existence of significant differences between MM and LM, as well as between MP and LP (Figure S1). The model included fixed effects for side (medial vs. lateral) and receptor type, with hemisphere treated as a random effect. The following Eq. (3) was used for analysis:

3 $$\:{D}_{l,r,h}=\:{\alpha\:}_{0}+\:{\alpha\:}_{1}{L}_{s}+\:{\alpha\:}_{2}{R}_{r}+\:{\alpha\:}_{3}{L}_{s}{R}_{r}+\:{\beta\:}_{1}{H}_{h}\:\:\:\:$$

where *D* is the receptor density, *S* is the side, *R* is the receptor type, and *H* is the hemisphere. The same analysis was applied separately to the magnocellular and parvocellular compartments.

#### Quantification of excitatory/inhibitory (E/I) and ionotropic/metabotropic ratios

The densities of the receptors for the principal excitatory neurotransmitter glutamate and the principal inhibitory neurotransmitter GABA are considerably higher than those of the receptors for modulatory neurotransmitters. Thus, we performed two analyses to characterise the ratio of receptor densities between excitatory and inhibitory neurotransmitter receptors within the entire LGN and V1 (i.e., after averaging laminar densities in each region). For the first measurement (E/I_G−G_ ratio), we focused on the receptors for glutamate and GABA. For the second measurement (E/I_mod_), we were interested in the ratio between the excitatory and the inhibitory receptors to which the modulatory neurotransmitters acetylcholine, noradrenalin, serotonin, and dopamine can bind (Table 1). In both cases (E/I_G−G_ and E/I_mod_), the ratio was computed by dividing the sum of the mean densities of excitatory receptors by the sum of the mean densities of inhibitory receptors.

4 $$\:{E/I}_{G-G}=\:\frac{AMPA+kainate+NMDA}{{GABA}_{A}+\:{GABA}_{B}+\:{GABA}_{A}/BZ}\:\:\:$$

5 $$\:{E/I}_{mod}=\:\frac{{{M}_{1}+{M}_{3}+{\alpha\:}_{4}{\beta\:}_{2}+\alpha\:}_{1}+\:{5HT}_{2}+\:{D}_{1}}{{M}_{2}+{\alpha\:}_{2}+\:{5HT}_{1A}}\:\:\:$$

We also sought to identify possible differences between the LGN and V1 in neurotransmission mediated by ionotropic and metabotropic receptors. Thus, to compute the ratio between ionotropic and metabotropic receptors (I/M), we divided the sum of the densities of all ionotropic receptors by the sum of the densities of all metabotropic receptors following Eq. 6.

6 $$\:I/M=\:\frac{AMPA+kainate+NMDA+{GABA}_{A}+{GABA}_{A}/BZ\:+\:{\:\alpha\:}_{4}{\beta\:}_{2}}{{GABA}_{B}+\:{M}_{1}+{M}_{2}+{M}_{3}+{\alpha\:}_{1}+{\alpha\:}_{2}+{5HT}_{1A}+\:{5HT}_{2}+\:{D}_{1}}$$

Finally, we performed paired t-tests to determine whether the LGN and V1 differ significantly from each other in their E/I_G−G_, E/I_mod_, and/or I/M ratios.

## Results

### Receptor architecture of the macaque LGN

**Fig. 3:**
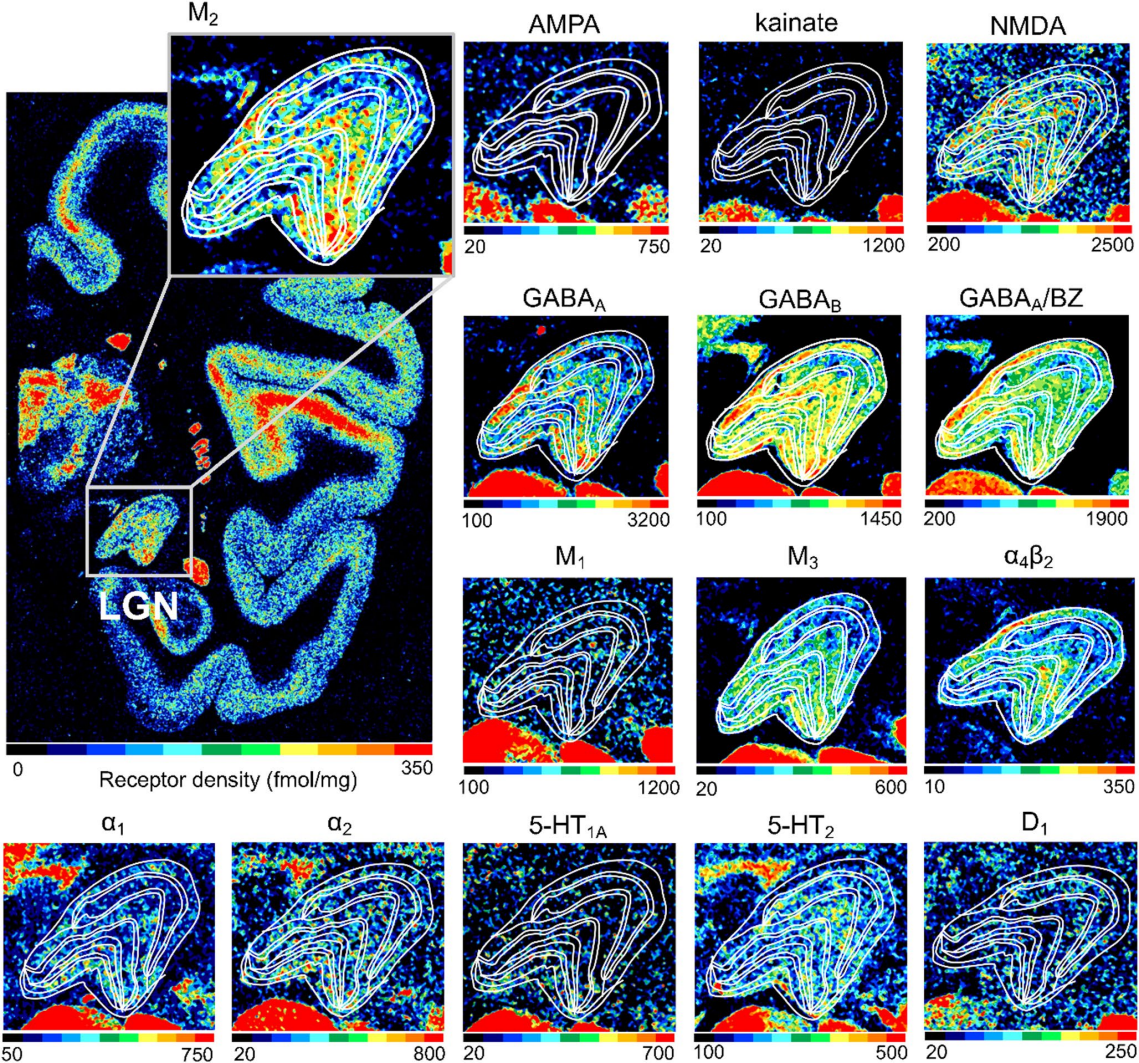
**Colour coded images showing the distribution of the analysed receptor types in the macaque LGN**. An entire coronal section taken approximately from Bregma level  -15.75 mm according to Paxinos et al. (2023) and +7 mm according to Saleem and Logothetis (2012) of a macaque monkey brain is shown for the M_2_ receptor, and the square highlights the LGN, which is also shown magnified. Colour coding indicates the density of each receptor with blue/green coding for low and orange/red coding for high densities. The density range (in fmol/mg protein) of each receptor type is specified under its respective colour scale. White lines indicate the borders among layers

Distribution of receptors for the classical neurotransmitters glutamate, GABA, acetylcholine, noradrenaline, serotonin, and dopamine in the macaque LGN is heterogeneous across receptor types, since some receptor types (e.g. GABA_B_ and GABA_A_/BZ) exhibit very high densities, while other receptor types (e.g., AMPA, 5-HT_1A_ or D_1_) exhibit very low level densities (Fig. 3).

**Fig. 4:**
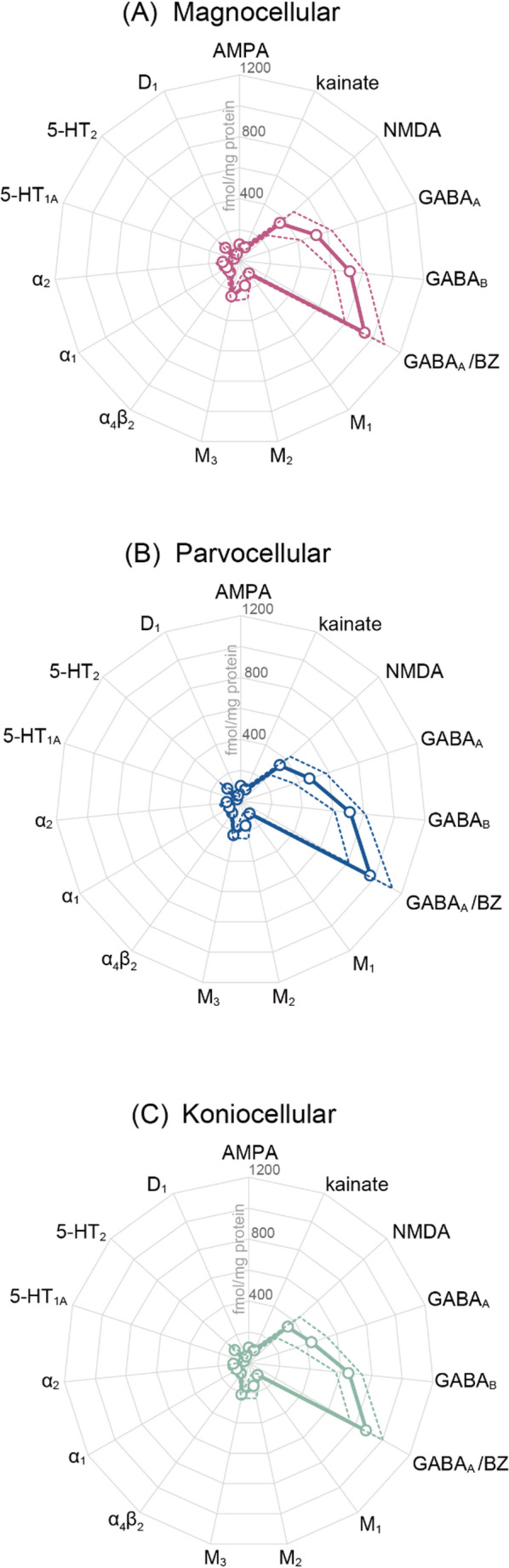
**Receptor fingerprints of the macaque LGN layers**. These radar plots depict densities of receptors, which are averaged among brains and provided in fmol/mg protein. (**A**) magnocellular, magenta, (**B**) parvocellular, blue, (**C**) koniocellular, green. Open circles and solid lines indicate the receptor density averaged across hemispheres, whereas dotted lines represent ± 1 standard deviation. Actual numbers used to generate this plot are included as supplementary data (Table S1). To facilitate the comparison of receptor types with low absolute densities, in Figure S3, we also present the fingerprints in the format with a log scale, where the unit is log^10^(fmol/mg protein)

Simple visual inspection revealed differences in receptor densities at the laminar level. For example, the koniocellular layers have a lower density of GABAergic receptors than do the magno- or parvocellular layers, and the same holds true for the M_3_ receptor (Fig. 3). However, when we visualised the densities as receptor fingerprints (Fig. 4; see also Table S1 for the original data), we found that the overall shape of receptor fingerprints is comparable across the three different types of layers, and that the fingerprints of the parvocellular and koniocellular layers were slightly smaller than that of the magnocellular layers. Parvocellular layer 3 presented the highest and lowest absolute densities measured within the LGN, with 845 fmol/mg protein of GABA_A_/BZ binding sites and 24 fmol/mg protein of 5-HT_1A_ receptors. Thus, since the analysed receptor types differ by a factor of 35 in their overall densities, we also generated receptor fingerprints using identical data, but with log scale (Figure S3), in which layer differences in receptor types with lower baseline density can be more identifiable. Again, we found that the fingerprints of parvocellular and koniocellular layers were only slightly smaller than that of the magnocellular layers. Indeed, while the omnibus test using the linear mixed model analysis revealed a significant main effect of layer type (*P* = 0.0001), it did not reveal any significant interaction between layer types and receptor types (*P* = 0.9977). We note, however, that the post-hoc test showed that the receptor density in magnocellular layers was significantly higher than in the parvocellular or koniocellular layers (*P* = 0.0004 and 0.0001, respectively).

Based on visual inspection, some receptors differ in density between the medial and lateral sides of the LGN (Fig. 3). Linear mixed model analysis for receptor density data extracted from the medial and lateral sides of the magnocellular and parvocellular compartments (Figure S1A) demonstrated that the mean receptor density was significantly higher in LP than in MP (*P* = 0.03268), though no global difference was found between LM and MM (*P* = 0.1269). However, the post-hoc analysis of the parvocellular compartments revealed no significant differences across receptor types. This indicates that the significant global difference arose from consistently but modestly higher receptor densities in the lateral compared with the medial parvocellular compartment, which did not reach statistical significance at the level of individual receptor types, as reflected by the overall similarity in receptor fingerprints of LP and MP (Figure S1B-E).

### Receptor architecture of macaque V1

**Fig. 5:**
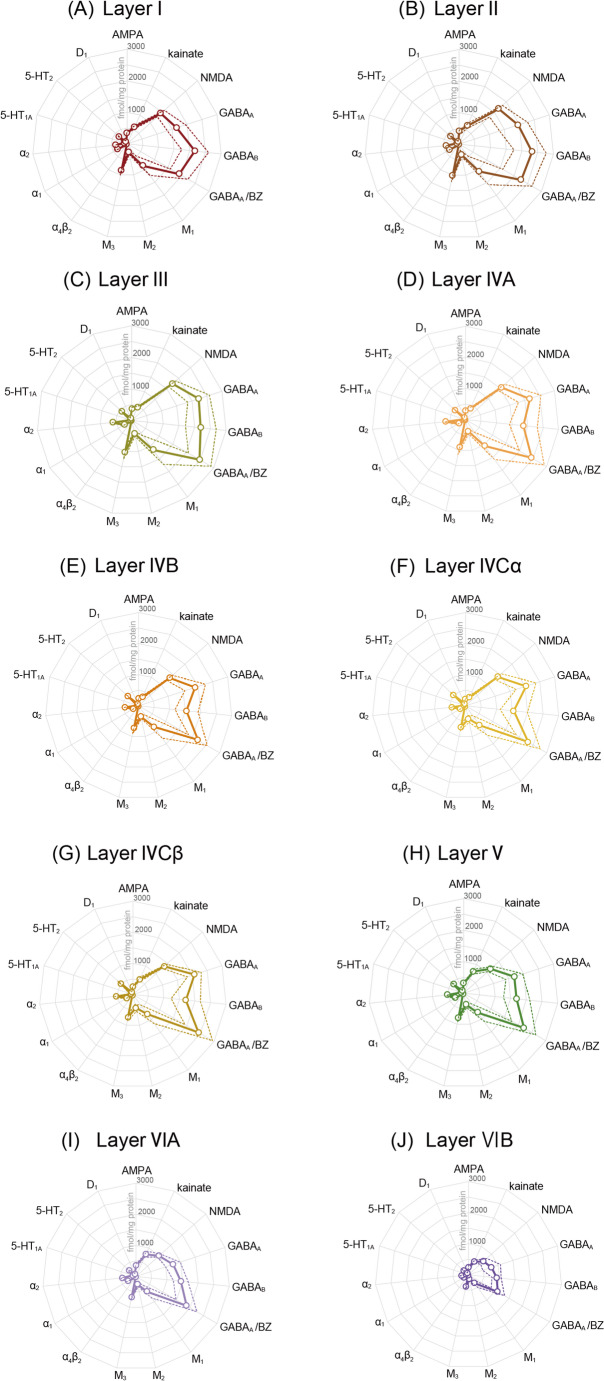
**Receptor fingerprints of macaque V1 layers I (A), II (B), III (C), IVA (D), IVB (E), IVCα (F), IVCβ (G), V (H), VIA (I), and VIB (J)**. Conventions are identical to those used in Fig. 4. Actual numbers used to generate this plot are included as supplementary data (Table S2). To facilitate the comparison of receptor types with low absolute densities, in Figure S4, we also present the fingerprints in the format with a log scale, where the unit is log^10^(fmol/mg protein)

We quantified the densities of the analysed receptors in each cytoarchitectonic layer of the macaque V1 (Figure S2), and also present this data as absolute receptor fingerprints (Fig. 5; Table S2), and as fingerprints with log scale (Figure S4). The shape of the absolute fingerprints depicted in Fig. 5 highlights the large difference in expression levels among receptor types, which spans a factor of approximately 100, given the 2482 fmol/mg protein GABA_A_/BZ and the 22 fmol/mg protein α_4_β_2_ densities present in layer III. These two values represent the highest and lowest values measured within the layers of V1, respectively. Although each receptor shows a specific laminar distribution pattern within V1, for most types we found higher densities in the supragranular than in the infragranular layers, whereby the local maximum was generally located in layer III. The kainate, nicotinic, and 5-HT_1A_ receptors constitute notable exceptions, due to their higher values in the infragranular than the supragranular layers. Layer IV was generally characterised by higher densities than those measured in layer VI, with the exception of the AMPA, kainate, and 5-HT_1A_ receptors, where the opposite holds true.

The analysed receptors also present a heterogeneous distribution throughout layer IV of V1. In some cases (e.g., AMPA, kainate, GABA_A_/BZ, and α_1_), layers IVA and IVCβ contain higher densities than do IVB and IVCα, and layer IVB generally shows lower densities than those measured in IVCα. In contrast, the densities of α_4_β_2_, 5-HT_1A_, 5-HT_2_, and D_1_ receptors are higher in layers IVCα and IVCβ than in IVA or IVB. Finally, NMDA receptors present comparable densities in layers IVB-IVCβ, which are clearly lower than those in IVA.

### Comparison of LGN and V1 receptor architecture

The receptor fingerprints of LGN layers and of V1 layers differ in size and shape (Figure S5), indicating differences not only in the absolute density of receptors contained in each of these two regions, but also in the balance between receptors that is typical for each one of them. Concerning differences in size, we found the receptor fingerprints of V1 layers to be considerably larger than those of LGN layers, indicating generally higher densities in V1 than in the LGN. This is particularly obvious when comparing the fingerprints depicting the absolute receptor densities (Figure S5). Indeed, receptor densities were two (M_2_, 5-HT_1A_, and D_1_ receptors) to 8 times (M_1_ receptors) higher in V1 than in the LGN. Differences in the shape of LGN and V1 fingerprints revealed striking contrasts concerning relative expression levels of the receptors for acetylcholine, in particular of the nicotinic α_4_β_2_ receptors, as well as variations in the different receptor types (i.e., excitatory vs. inhibitory and ionotropic vs. metabotropic).

#### The nicotinic cholinergic receptor

In general terms the balance between acetylcholine receptor densities and those of the other receptors varied between the LGN and V1. Interestingly, this did not hold true for the acetylcholine muscarinic M_3_ receptor. The density of M_1_ receptors was comparable to that of AMPA, kainate, α_1_, α_2_, and 5-HT_2_ receptors in the LGN, but was twice as high in V1. The opposite situation was observed for the M_2_ receptor. It presented comparable densities to AMPA, kainate, α_2_, and 5-HT_2_ receptors in V1, but not more than half their amount in the LGN.

**Fig. 6:**
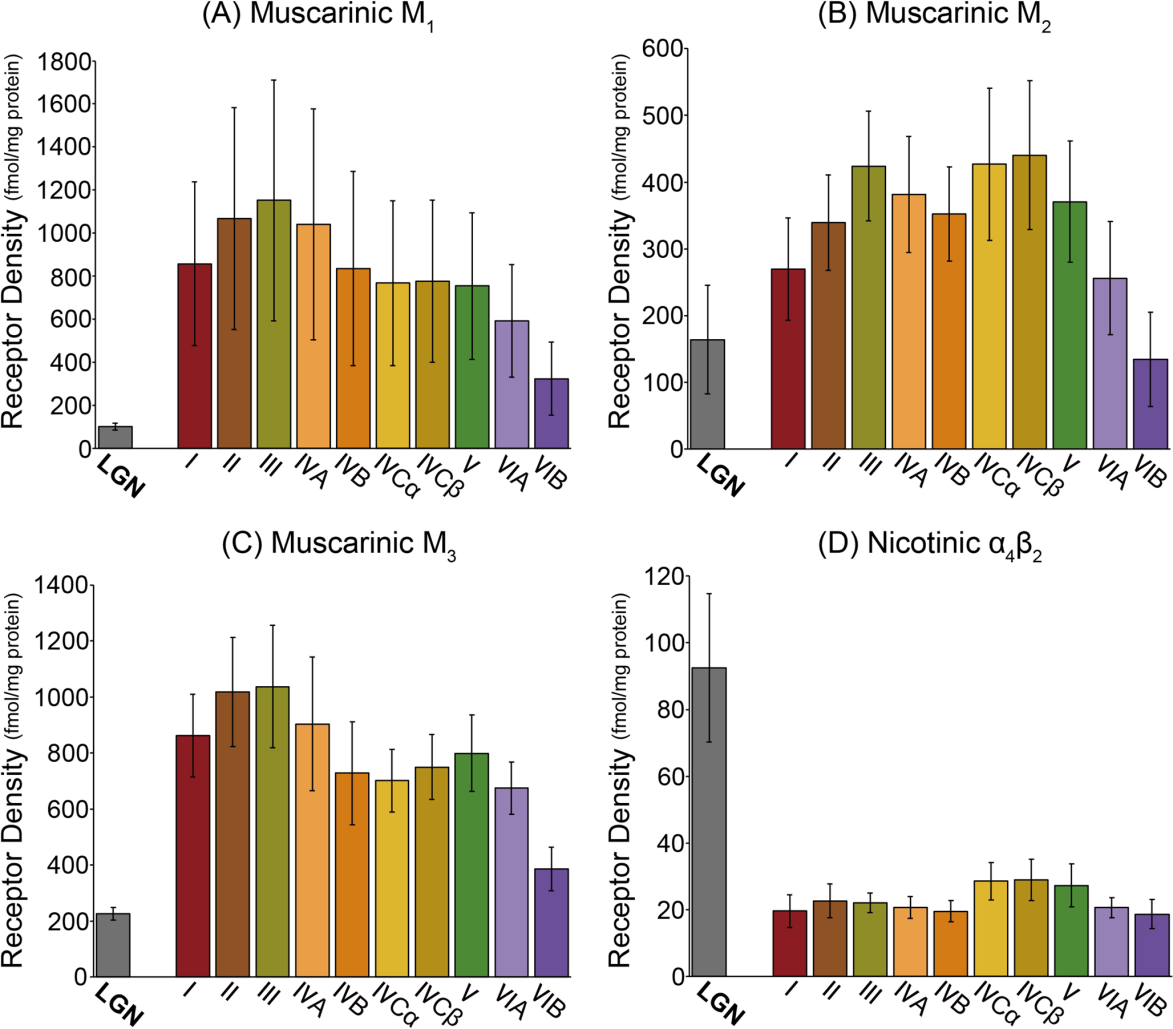
**Densities of receptors for acetylcholine in the LGN (grey bar) and in the layers of the V1 (coloured bars)**. (**A**) Muscarinic M_1_ receptors, (**B**) muscarinic M_2_ receptors, (**C**) muscarinic M_3_ receptors, and (**D**) nicotinic α_4_β_2_ receptors. Each bar represents the average data of each ROI. Error bars depict ± 1 standard deviation

The nicotinic α_4_β_2_ receptors display a unique constellation, since they are the only receptor type present at a higher density in the LGN than in V1. While the overall density of receptor density in α_4_β_2_ is low, this inverse relationship unique to this receptor suggests that this receptor may have a distinctive contribution to visual processing in the LGN. Given this striking finding regarding the nicotinic α_4_β_2_ receptor and the fact that, despite their overall relatively low expression levels, muscarinic receptors presented both the smallest and largest differences in densities between the LGN and V1. Fig. 6 depicts a comparison of receptor density of acetylcholine receptors between these two brain regions.

We assessed the statistical significance of the relationship between receptor densities in the LGN and in V1 by means of a stepwise linear mixed-effect model analysis using data from all 15 receptors and two ROIs (LGN and V1). The omnibus test (analysis of variance, ANOVA) based on a linear mixed-effect model showed statistically significant interaction effects between receptor types and ROIs (*P* = 0.0000). Subsequent simple effect tests show that all receptor types except for α_4_β_2_ showed a significantly higher density in the V1 than the LGN (*P* = 0.0000 in all cases). In contrast, α_4_β_2_ showed a significantly higher density in the LGN than V1 in simple effect tests (*P* = 0.0000). These results showing significant interaction between receptor types and ROIs suggest that nicotinic α_4_β_2_ is a unique receptor regarding its relatively higher density in the LGN than in V1.

#### Comparison of receptor density ratios in the LGN and V1

**Fig. 7:**
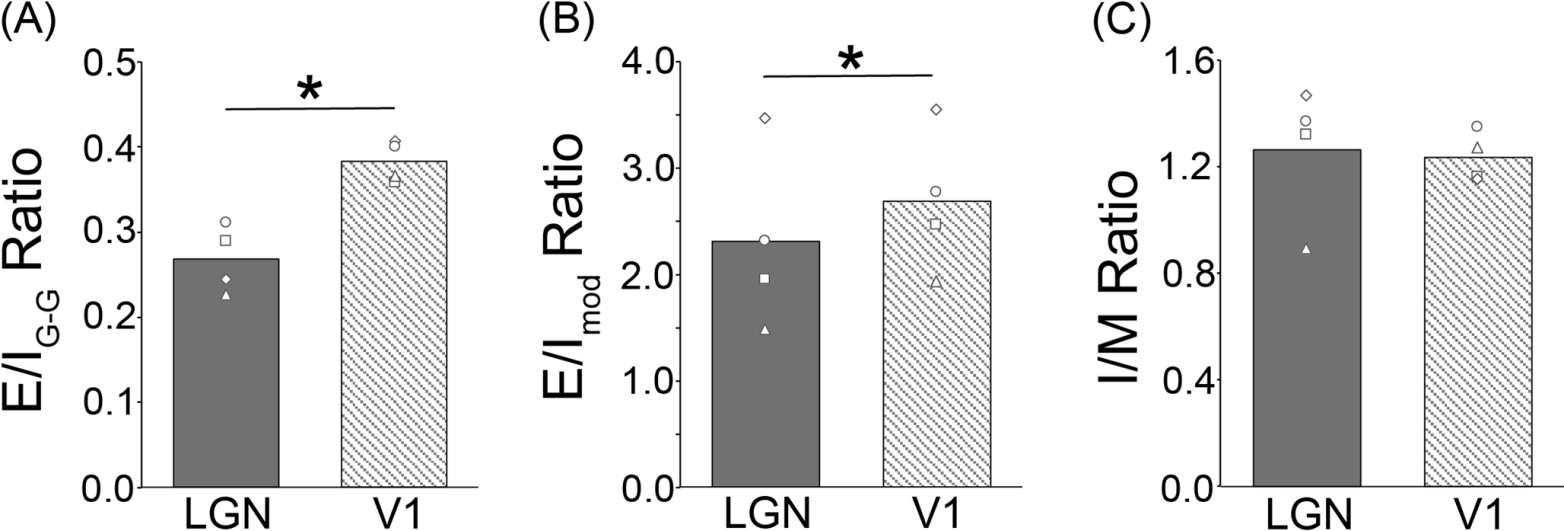
**Excitatory/inhibitory (E/I) and ionotropic/metabotropic (I/M) ratios in the LGN and V1**. (**A**) Ratio between receptors for the excitatory and inhibitory neurotransmitters glutamate and GABA, respectively (E/I_G−G_). It results from dividing the sum of the densities of all receptors for glutamate (AMPA, kainate, and NMDA) by the sum of the densities of all GABAergic binding sites (GABA_A_, GABA_A_/BZ, and GABA_B_). **(B)** Ratio between the excitatory and inhibitory receptors for the modulatory neurotransmitters acetylcholine, noradrenalin, serotonin, and dopamine (E/I_mod_). It results from dividing the sum of the densities of all their excitatory receptors (M_1_, M_3_, α_4_β_2_, α_1_, 5-HT_2_, and D_1_) by the sum of the densities of all their inhibitory receptors (M_2_, α_2_, and 5-HT_1A_). **(C)** Ratio between the ionotropic and metabotropic receptors (I/M ratio). It results from dividing the sum of the densities of all the ionotropic receptors (AMPA, kainate, NMDA, GABA_A_, GABA_A_/BZ, and α_4_β_2_) by the sum of the densities of all the metabotropic receptors (GABA_B_, M_1_, M_2_, M_3_, α_1_, α_2_, 5-HT_1A_, 5-HT_2_, and D_1_). In each plot, the bars indicate the mean ratio of the LGN (dark grey) and V1 (light grey) across hemispheres, while open symbols (circles, triangles, squares, diamonds) depict the data from individual hemispheres. Each ROI (LGN and V1) includes all layers. A larger number indicates a relatively larger density of excitatory receptors over that of inhibitory receptors, or of ionotropic receptors over that of metabotropic receptors

We evaluated the ratio of receptor density for excitatory and inhibitory receptors (E/I ratio), to better understand the functional roles of the LGN and V1 during visual processing (Fig. 7) and first focused only on receptors for glutamate and GABA, the primary excitatory and inhibitory neurotransmitter receptors, respectively, in the adult brain. We found that V1 showed a higher E/I_G−G_ ratio compared with the LGN (paired t-test, t_3_ = -5.34, *P* = 0.01; Fig. 7). We performed a comparable analysis, though this time with a focus on the excitatory and inhibitory receptors for modulatory neurotransmitters. We found that V1 exhibits a statistically significantly higher E/I_mod_ ratio compared with the LGN (paired t-test, t_3_ = -3.75, *P* = 0.03).

Besides the differences mentioned above, which are relevant for the balance between excitatory and inhibitory neurotransmission, we observed region specific patterns in the relationship between ionotropic and metabotropic receptors. For example, in the LGN, GABA_B_ receptors are present at an intermediate value between those of the GABA_A_ receptor and the GABA_A_/BZ binding sites. In contrast, layer IV of V1 contains a lower density of the GABA_B_ receptor than of the GABA_A_ receptor or the GABA_A_/BZ binding sites, indicating that the LGN and V1 differ in their balance of metabotropic and ionotropic GABAergic receptors. However, the statistical analysis revealed no significant differences between the LGN and V1 concerning their I/M ratios (paired t-test, t_3_ = 0.19, *P* = 0.86).

## Discussion

We applied in vitro receptor autoradiography to evaluate the receptor architecture of the macaque LGN and achieve a better understanding of the organisation of neurotransmitter systems supporting early visual processing in primates. We found considerable variations in expression levels across receptors in the LGN and in V1, though they were larger in V1 than in LGN. Receptor fingerprints of the LGN differed in shape and size from those of V1. The nicotinic α_4_β_2_ receptor presents a unique constellation since it is the only receptor type with a higher density in the LGN than in V1. Finally, we found that the LGN and V1 have different ratios between excitatory and inhibitory receptors, though not between ionotropic and metabotropic receptors.

### Laminar differences

We found the magnocellular LGN layers to contain significantly higher receptor densities than the koniocellular or parvocellular layers. Given that signals from each LGN sublayer contribute to subsequent cortical visual information processing in different ways (Maunsell et al. 1990), the results suggest that signals from different sublayers are subject to different neurochemical controls.

In macaque V1, most receptors presented higher densities in the supragranular than in the infragranular layers, a pattern consistent with the distribution of synapses (O’Kusky and Colonnier 1982), as also described for the human brain (Palomero-Gallagher and Zilles 2019). The higher receptor density in layer IV compared with layer VI is likely related to differences in synaptic density (O’Kusky and Colonnier 1982; Wilson 1989). However, since the association between receptor and synaptic densities is not generalisable across all brain regions (Plaza-Alonso et al. 2025), future studies must determine the relationship between synaptic and receptor measurements in V1.

### Comparison of the medial and lateral sides of the LGN

We also sought to determine the existence of density differences between the medial and lateral sides of the LGN. The global test reached significance only for the parvocellular compartment, encompassing the parvocellular layers and adjacent koniocellular layers, while none of the post hoc tests were significant. This suggests that the effect was driven by modestly higher receptor densities in the lateral versus medial parvocellular compartment, insufficient to reach statistical significance for individual receptor types, as reflected in the overall similarity of LP and MP receptor fingerprints (Figure S1D-E). The medial and lateral LGN correspond to different visual field representations, with the medial and lateral LGN representing the lower and upper visual fields, respectively (Malpeli and Baker 1975). Previous studies have reported asymmetry of retinal ganglion cell density (Curcio and Allen 1990; Watson 2014) and V1 surface area (Kupers et al. 2022; Himmelberg et al. 2023) between lower and upper visual field representations. Although to our knowledge asymmetric cell density in the LGN has not been previously reported, it is plausible that the medial and lateral LGN have different anatomical characteristics, given that asymmetry has been observed in both retinal ganglion cells and V1.

### Related studies

Our results are consistent with previous studies focusing on a small number of specific receptor types in the LGN. For example, a receptor autoradiography study by Shaw and Cynader (1986) also reported that GABA_A_ and GABA_A_/BZ presented the highest densities in macaque LGN among the eight receptors they investigated. In situ hybridisation and autoradiography studies have shown that the macaque LGN contains a higher NMDA receptor density compared to that of the AMPA receptor (Jones et al. 1998; Ibrahim et al. 2000), and our receptor fingerprint also showed this trend (Fig. 4). Furthermore, the relatively higher nicotinic receptor density in the LGN than in V1, which will be discussed in detail further below, was also reported in a previous study (Cimino et al. 1992).

Rodent studies also showed a high density of GABA receptors in the LGN (Chu et al. 1990; Li et al. 2003; Neto et al. 2006), consistent with the macaque data. In contrast, our results show species differences concerning the muscarinic receptors; whereas in rodent LGN M_2_ receptor density is higher compared to other muscarinic receptors (Plummer et al. 1999), in the macaque we found the M_3_ receptor to present the highest density. Given the evolutionary difference in the role of the LGN between rodents and primates in visual processing (Kaas et al. 2022), some inter-species differences in the LGN are likely to exist. Evaluating how these receptor architecture differences between primates and rodents are related to primates’ specialisation of certain visual functions is an important topic for future investigations.

### Similarities and differences in E/I ratios in the LGN and V1

One similarity between the LGN and V1 is that GABAergic receptors were present at higher densities than those of the other examined receptor types, as also described for cortical areas spread throughout the macaque (Impieri et al. 2019; Rapan et al. 2021, 2022, 2023; Niu et al. 2020, 2021, 2024; Kotter et al. 2001; Bozkurt et al. 2005), and human brains (Zilles and Palomero-Gallagher 2017b). Extremely low 5-HT_1A_ and D_1_ densities constitute further similarities between the LGN and V1. V1 has one of the lowest 5-HT_1A_ densities measured throughout the macaque cortex (Froudist-Walsh et al. 2023). Additionally, the LGN and V1 contain comparable AMPA and M_2_ receptor densities, whereas extrastriate cortical areas contain higher AMPA than M_2_ receptor densities (Impieri et al. 2019; Rapan et al. 2021, 2022, 2023; Niu et al. 2020, 2021, 2024; Kotter et al. 2001; Bozkurt et al. 2005), and the same applies to the human brain (Palomero-Gallagher and Zilles 2019).

We found that the LGN and V1 differ in terms of the balance between their excitatory and inhibitory receptor densities (both for E/I_G−G_ and E/I_mod_ ratios), though not in their I/M ratio, suggesting that both brain regions could integrate fast, immediate responses with longer-term modulation, allowing dynamic and flexible regulation of neural circuits (Linders et al. 2022). The lower E/I_G−G_ and E/I_mod_ ratios in LGN than in V1 further confirm that it is not a simple relay station for retinal input to the cortex (Sherman and Koch 1986; Fisher et al. 2017). They may reflect the involvement of retinal *and* non-retinal circuits in early visual processing (Alitto and Usrey 2008), and highlight the importance of modulatory neurotransmission in the downstream processing of visual input. Specifically, the LGN’s E/I_mod_ ratio could constitute the molecular underpinning for surround suppression, where neuronal response is suppressed by the presence of visual input outside classical receptive fields (Li et al. 2024), an essential LGN function supporting the cortical processing necessary to sharpen visual information or perform contrast gain control (Sherman and Koch 1986; Bonin et al. 2005).

The release of glutamate at the retino-fugal synapse is subject to inhibitory modulation via presynaptically located GABA_B_ and 5-HT_1_ receptors (Chen and Regehr 2003; Seeburg et al. 2004; Reggiani et al. 2023; Yoshida et al. 1984). The excitatory feedback from V1 layer VI neurons constitutes the major source of non-retinal input to the LGN (Tsumoto et al. 1978; Jones et al. 2012). Non-retinal input also arises from other regions spread throughout the brain and is mediated by various neurotransmitters. The macaque LGN receives a sparse and homogeneous serotonergic innervation (Wilson et al. 1995; Wilson and Hendrickson 1988; Pasik et al. 1988). Serotonin has a generally dampening effect in the LGN, either directly via the presynaptic inhibitory 5-HT_1_ receptors (Chen and Regehr 2003; Seeburg et al. 2004; Reggiani et al. 2023; Yoshida et al. 1984), or indirectly via excitatory 5-HT_2_ receptors on intrageniculate interneurons (Yoshida et al. 1984). Some studies described an extremely sparse noradrenergic innervation to the LGN (Perez-Santos et al. 2021; Morrison and Foote 1986), while others reported its total absence (Bowden et al. 1978). Interestingly, noradrenaline enhances the responsiveness of neurons in the LGN via activation of both noradrenergic α_1_ and β receptors (Nakai and Takaori 1974; Rogawski and Aghajanian 1980). Further sources of non-retinal input to the LGN include the intrinsically located interneurons as well as GABAergic neurons in the thalamic reticular nucleus, perigenicular nucleus and pretectum (Fisher et al. 2017; Kimura 2014; Alitto and Usrey 2008; Gabbott et al. 1986; Montero et al. 2001; Montero and Zempel 1986; Hendrickson et al. 1983; Sabbagh et al. 2021; Uhlrich and Cucchiaro 1992; Bickford et al. 2000). Thus, our findings highlight the critical role of inhibitory neurotransmission in the LGN in shaping downstream visual processing. Furthermore, this effect is not mediated solely by GABA, but also by the binding of modulatory neurotransmitters to their inhibitory receptor types.

### Cholinergic neurotransmission in visual processing: unique nicotinic receptor constellation

A notable finding of this study was that the LGN generally has lower receptor densities than V1, except for the nicotinic α_4_β_2_ receptor, which was four times more abundant in the LGN. This result is consistent with previous studies in rodents (Clarke et al. 1985; Palomero-Gallagher et al. 2008; Cremer et al. 2011, 2015), macaques (Cimino et al. 1992), and humans (Palomero-Gallagher and Zilles 2018; Adem et al. 1988; Palomero-Gallagher et al. 2015). Expanding on this finding, in humans, the thalamus is the brain structure with the highest density of nicotinic α_4_ꞵ_2_ receptors, and visual thalamic nuclei presented the second highest nicotinic density after that of the prefrontal thalamic group (Garibotto et al. 2020).

The muscarinic M_2_ receptor is among the most highly expressed in the LGN and is the only receptor besides the nicotinic α_4_β_2_ with a relatively higher mean density there than in many cortical areas across the macaque brain. The brains analysed here are the same ones that were used in previous studies to characterise the receptor architecture of the macaque frontal (Rapan et al. 2023), motor (Rapan et al. 2021), parietal (Impieri et al. 2019; Niu et al. 2020, 2021, 2024), and occipital (Rapan et al. 2022) cortex, thus enabling this direct comparison of the reported densities.

The basal forebrain is the primary source of cholinergic input to the cortex (Zaborszky et al. 1999; Hajszan and Zaborszky 2002; Mesulam 1995; Selden et al. 1998), whereas the primate LGN, which presents the highest density of acetylcholine reactive terminals among all thalamic nuclei, receives its cholinergic input from the upper brainstem (Wilson et al. 1995; Uhlrich and Cucchiaro 1992; Heckers et al. 1992; Huerta-Ocampo et al. 2020; Steriade et al. 1988). Previous studies have shown that acetylcholine inputs from the pedunculopontine tegmental and the parabigeminal nuclei to the LGN are associated with the enhancement of neuronal response to the visual input (Sherman 1996; Uhlrich et al. 1995; Kobayashi and Isa 2002) and visuomotor control (Cui and Malpeli 2003), respectively.

The functional significance of muscarinic and nicotinic receptors in the visual system is well-studied in physiological studies. In V1, blockade of either receptor type with an antagonist reduced the neural response and sensitivity to visual stimuli (Herrero et al. 2017). Activation of nicotinic receptors in V1 increases the response of layer IV neurons to visual stimuli and reduces neuronal contrast threshold (Disney et al. 2007), whereas activation of muscarinic receptors reduces noise correlation and improves coding efficiency to visual stimuli (Minces et al. 2017; Goard and Dan 2009). An elegant PET study involving pharmacological manipulation of the cholinergic system demonstrated a region specific and differential role of muscarinic and nicotinic receptors in the modulation of visual stimuli (Mentis et al. 2001). The authors demonstrated that the modulatory effect of acetylcholine was predominantly mediated by muscarinic receptors in V1 and early visual areas, but by nicotinic receptors in the thalamus and inferior parietal lobule. Further, cholinergic activity via muscarinic receptors was associated with the improvement of signal-to-noise ratio and the modulation of visual attribute processing, and activation of the nicotinic receptors was interpreted as being responsible for modulating selective attention to the visual task (Mentis et al. 2001). Our results demonstrate that these effects cannot be explained by absolute differences in the expression levels of these two cholinergic receptor types, but by their different balance in the LGN and V1.

### Strengths and limitations of in vitro receptor autoradiography

While in vitro receptor autoradiography allows measurement of absolute neurotransmitter receptor densities, it does not reveal the specific cell types expressing them or distinguish between pre- and postsynaptic receptor localisation. Therefore, the focus of our study is to characterise the receptor architecture of the LGN at the mesoscopic level, rather than to build models of microcircuitry (Douglas and Martin 2004).

In addition, since the receptor autoradiography dataset analysed in this study has not yet been reconstructed in three-dimensional space, we were unable to perform a quantitative comparison with the existing neuroimaging dataset of macaque monkeys. If three-dimensional reconstruction became possible, one possible future venue is to compare with the representation of the visual field, which can be measured by fMRI (Brewer et al. 2002; Kolster et al. 2014; Arcaro and Livingstone 2017), to gain insight into whether there is functionally meaningful variation within each sublayer of the LGN.

Despite these interpretive limitations, this approach is vital for system neuroscience in several ways. First, it enables quantitative comparison of receptor fingerprints across different areas, providing essential insights into how brain areas can be categorised into specific networks and thereby improving the interpretation of neuroimaging studies (Zilles et al. 2015). Second, since this approach is broadly applicable across species, including humans, it facilitates understanding of the similarities and differences in brain areas across species (Rapan et al. 2022). Our results provide a strong foundation for understanding the architecture of the macaque LGN, which will be valuable for future comparative studies.

## Supplementary Information

Below is the link to the electronic supplementary material.

Supplementary Material 1

## Data Availability

The original data, including numbers of receptor densities used for generating figures shown in this manuscript, are provided in the Supplementary Tables (Tables S1 and S2) and have been made publicly available via the EBRAINS platform (https://doi.org/10.25493/VS8J-V0E).
