# Supplementary material for: Receptor architecture of the macaque lateral geniculate nucleus
Source: Brain Struct Funct. 2026 May 19;231(5):67. doi: 10.1007/s00429-026-03109-5 (PMC13186819; doi:10.1007/s00429-026-03109-5)
Supplement: Supplementary file 1 — Supplementary Material 1 [file 429_2026_3109_MOESM1_ESM.docx]

Receptor architecture of the macaque lateral geniculate nucleus

- Supplementary information –

**Authors:** Marina Saito^1,2,3,4)^, Lucija Rapan^5)^, Meiqi Niu^5)^, Ling Zhao^5,6)^, Sei-ichi Tsujimura^2)^, Hiromasa Takemura^1,7,8)*^ , Nicola Palomero-Gallagher^5,9)*^

**Affiliations:**

1) Division and Sensory and Cognitive Brain Mapping, Department of System Neuroscience, National Institute for Physiological Sciences, Okazaki, 444-8585 Japan

2) Faculty of Design and Architecture, Nagoya City University, Nagoya, 464-0083 Japan

3) Japan Society for the Promotion of Science, Tokyo, 102-0083 Japan

4) Laboratory for Imagination and Executive Functions, RIKEN Center for Brain Science, Wako, 351-0106 Japan

5) Institute of Neuroscience and Medicine (INM-1), Research Centre Jülich, Jülich 52425, Germany

6) Department of Psychology, School of Public Policy and Management, Nanchang University, Nanchang, 330031 China

7) The Graduate Institute of Advanced Studies, SOKENDAI, Hayama, 240-0115 Japan

8) Core for Spin Life Sciences, Okazaki Collaborative Platform, National Institutes of Natural Sciences, Okazaki, 444-8585 Japan

9) C. & O. Vogt Institute of Brain Research, Heinrich-Heine University Düsseldorf, Düsseldorf 40225, Germany

*These authors made equal senior contributions

**Corresponding authors:**

Marina Saito
Faculty of Design and Architecture

Nagoya City University

2-1-10, Kita Chikusa, Chikusa-ku, Nagoya, 464-0083, Japan
marina.nagoyasda.forward@gmail.com

Nicola Palomero-Gallagher

Institute of Neuroscience and Medicine (INM-1)

Research Centre Jülich

52425 Jülich, Germany

n.palomero-gallagher@fz-juelich.de

**
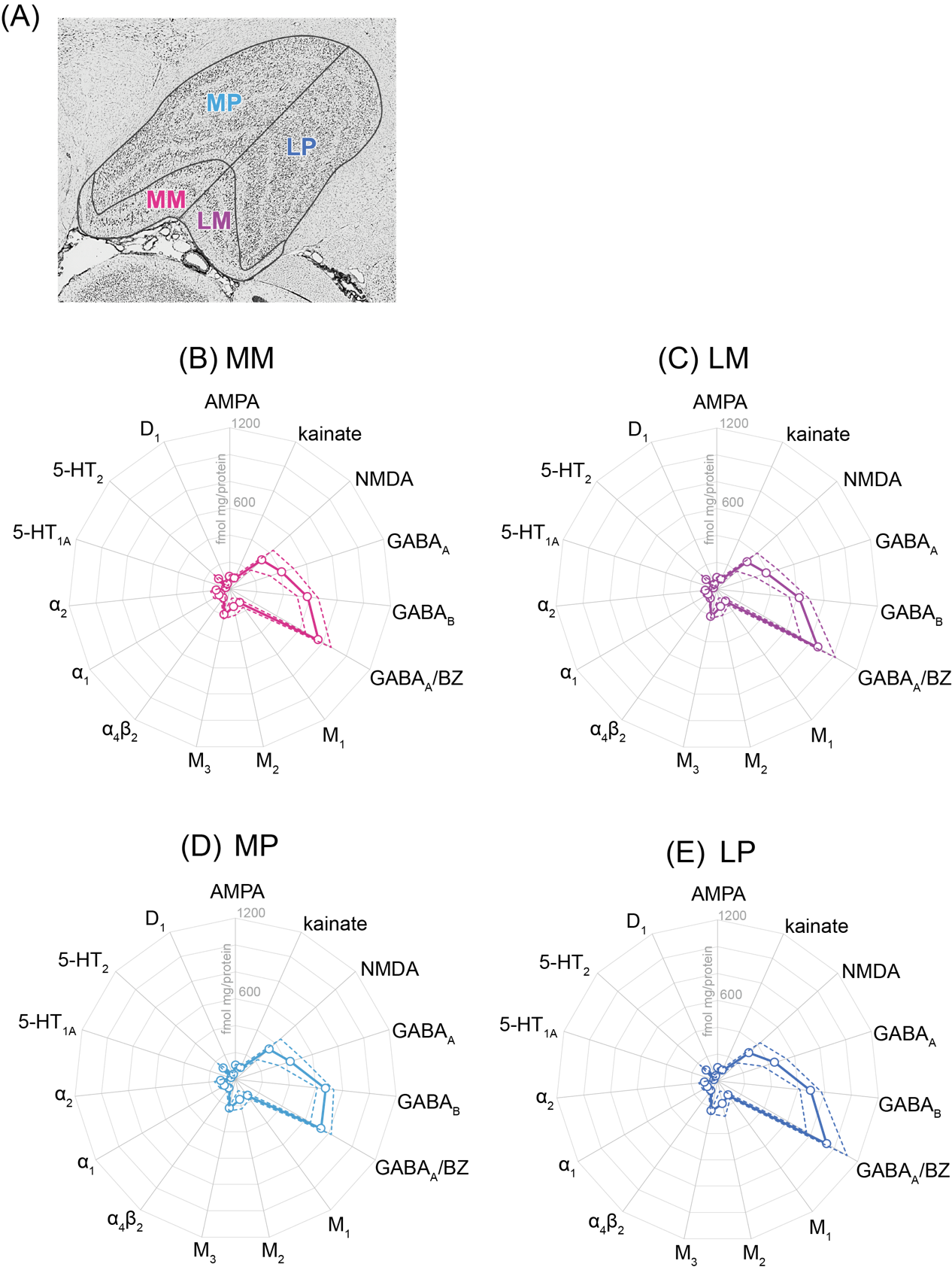
**

**Figure S1: Analysis of medio-lateral differences in receptor densities within the LGN. A.** Exemplary silver cell body-stained coronal section (the same one as shown in Figure 2: section #29; brain #rh11530) depicting the medial and lateral compartments defined within the LGN. Black contours depict the manually delineated borders between the medial and lateral portions of the LGN. Additionally, we differentiated between the magnocellular (and adjacent koniocellular) and parvocellular (and adjacent koniocellular) layers to form the magnocellular and parvocellular compartments, respectively. **B-E.** Receptor fingerprints of medial and lateral sides of the magnocellular compartment (MM and LM; panels B and C, respectively) and of the medial and lateral sides of the parvocellular compartment (MP and LP; panels D and E, respectively). Conventions are identical to those used in Figure 4.


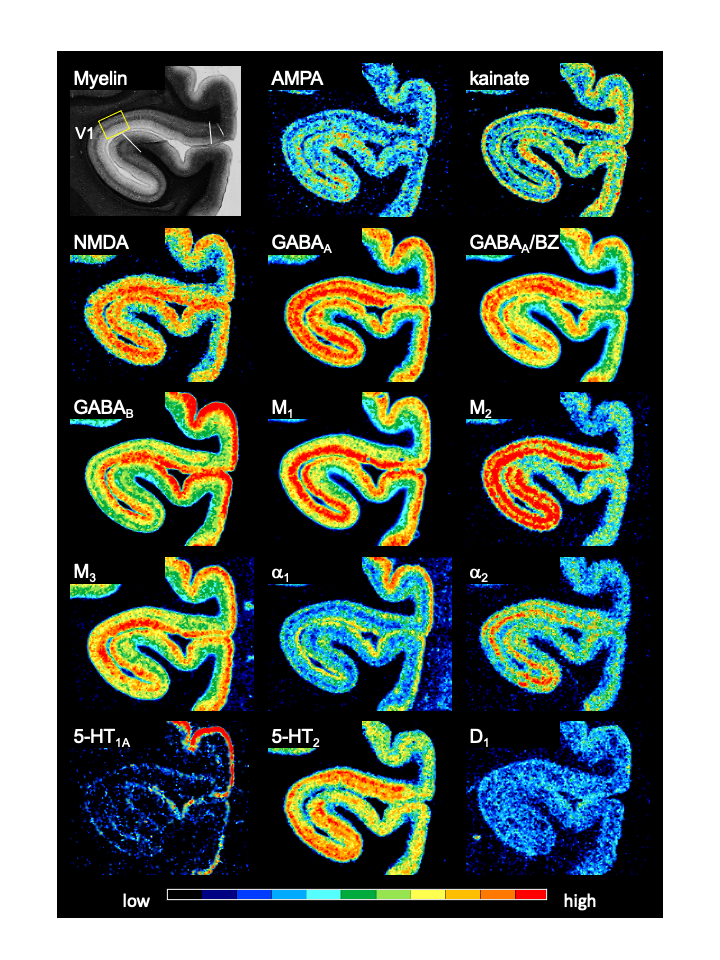


**Figure S2: Colour coded images showing the distribution of the analysed receptor types in the macaque V1.** Serial coronal sections through the calcarine sulcus at Bregma level -31.50mm according to Paxinos et al. (2023) and -5mm according to Saleem and Logothetis (2012), and which were processed for the visualisation of myelin sheaths or of the examined receptor types. The white lines in the myelin stained section indicate the position of the borders between V1 and V2. The yellow frame indicates the location from which receptor densities were extracted in the corresponding autoradiographs.


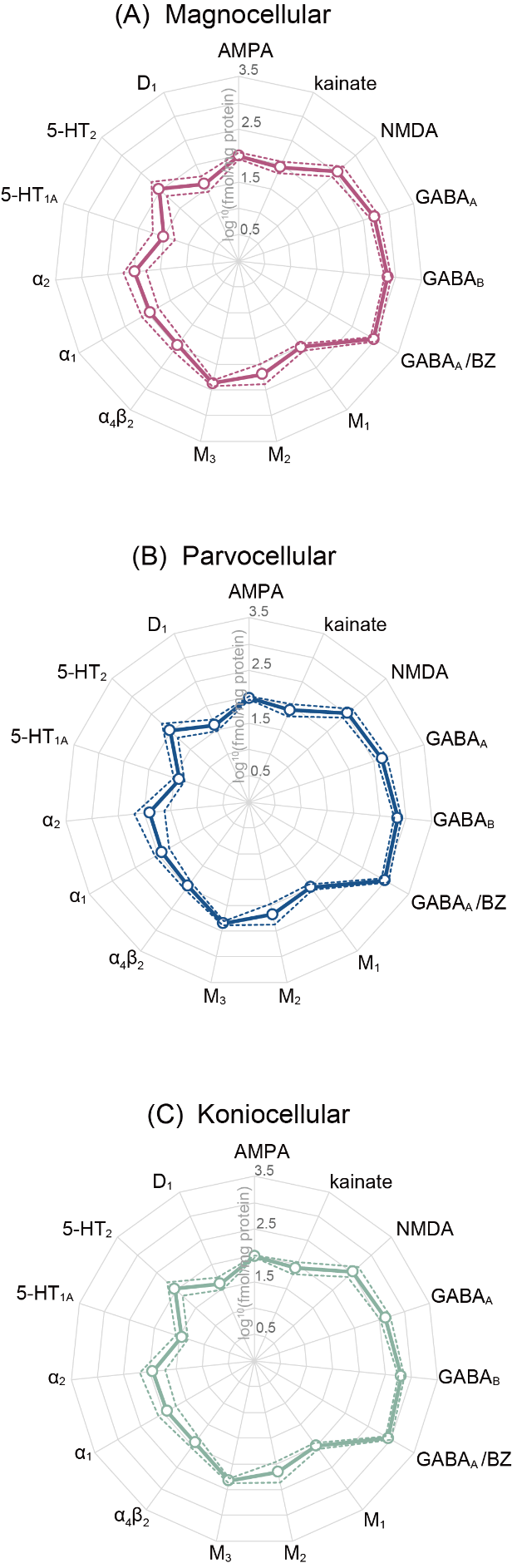


**Figure S3: Log-scale receptor fingerprint of the macaque LGN layers.** This figure visualises receptor data shown in Figure 4, but with a log-scale. The unit is log^10^(fmol/mg protein).


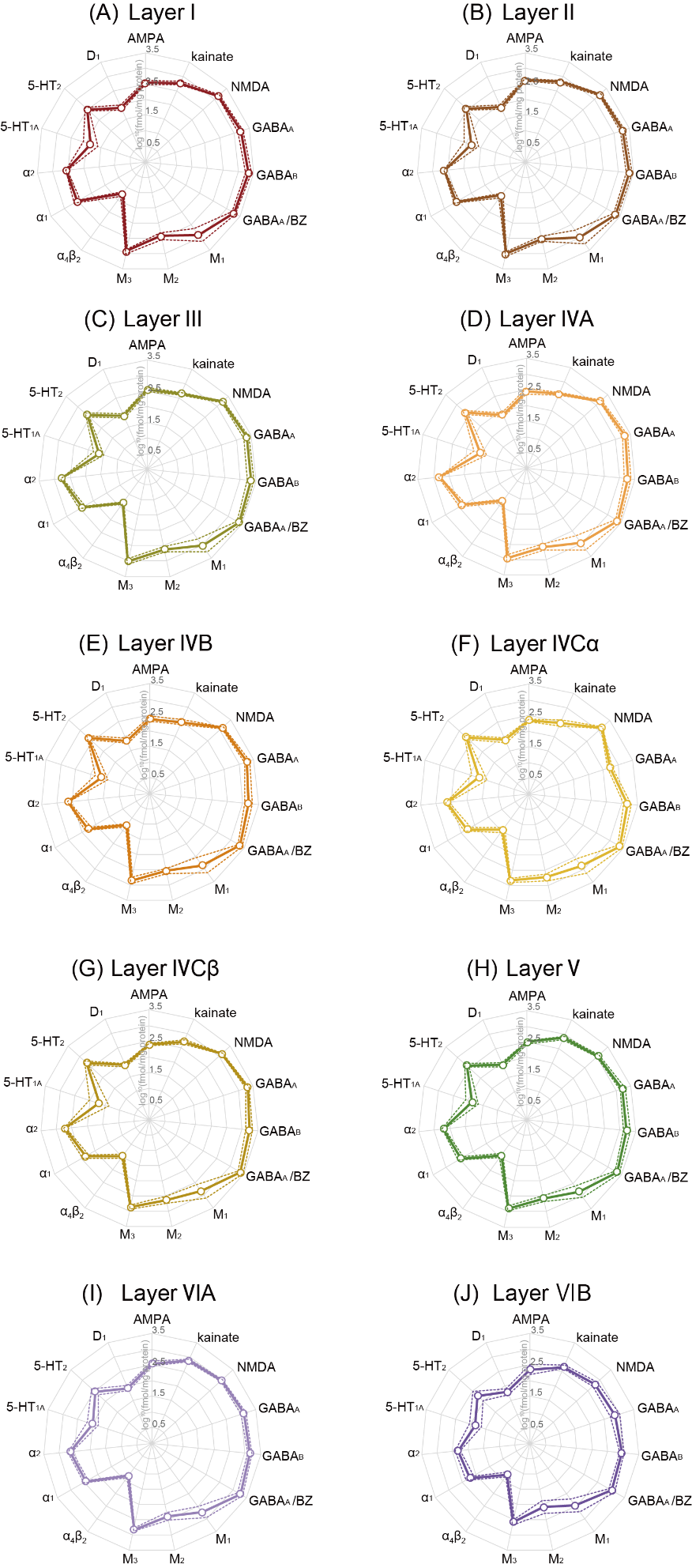


**Figure S4: Log-scale receptor fingerprint of the macaque V1 layers.** This figure visualises receptor data shown in Figure 5, but with a log-scale. The unit is log^10^(fmol/mg protein).


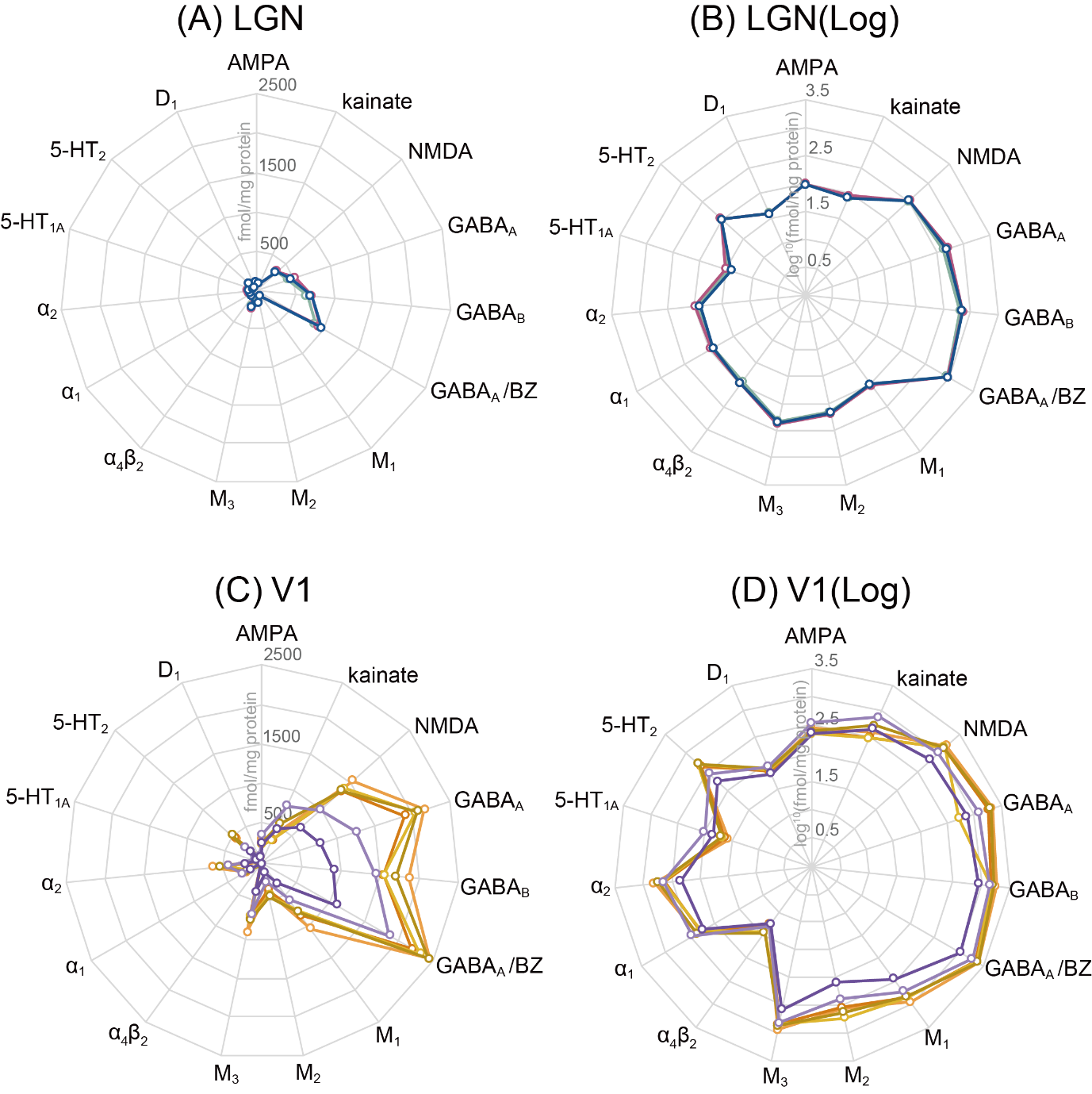


**Figure S5: Comparison of LGN and V1 fingerprints with identical scaling.** To facilitate a comparison of the receptor architecture of the LGN and V1, this figure summarises receptor data shown in Figures 4 and 5 and in Figures S3 and S4. Each colour depicts data from each LGN and V1 sublayer, using the same convention as Figures 4 and 5. Note, that the axis scaling of the absolute receptor fingerprints has been adjusted with respect to that shown in Figures 4 and 5. Here it is the same in both plots depicting the same unit format (i.e., both plots showing receptor densities in fmol/mg protein share one axis scaling and both plots showing receptor densities in log^10^(fmol/mg protein) also share the same axis scaling), thus facilitating visualisation of the differences in the size of the fingerprints of these two regions.

**Table S1: Mean and standard deviation (SD) of receptor density (in fmol/mg protein) in each layer of the LGN.**

|  |  | **AMPA** | **kainate** | **NMDA** | **GABA_A_** | **GABA_B_** | **GABA_A_/BZ** | **M_1_** | **M_2_** | **M_3_** | **α_4_ꞵ_2_** | **α_1_** | **α_2_** | **5-HT_1A_** | **5-HT_2_** | **D_1_** |
| --- | --- | --- | --- | --- | --- | --- | --- | --- | --- | --- | --- | --- | --- | --- | --- | --- |
| **M1** | Mean | 101 | 89 | 360 | 543 | 747 | 774 | 109 | 175 | 244 | 100 | 98 | 112 | 36 | 95 | 42 |
|  | SD | 15 | 27 | 106 | 128 | 98 | 109 | 18 | 72 | 34 | 28 | 42 | 46 | 15 | 57 | 16 |
| **M2** | Mean | 98 | 91 | 353 | 503 | 695 | 726 | 102 | 166 | 234 | 95 | 90 | 101 | 33 | 95 | 41 |
|  | SD | 11 | 23 | 123 | 82 | 119 | 188 | 18 | 92 | 34 | 31 | 41 | 45 | 14 | 65 | 16 |
| **P1** | Mean | 98 | 88 | 357 | 490 | 737 | 808 | 102 | 190 | 242 | 108 | 90 | 104 | 28 | 96 | 40 |
|  | SD | 10 | 27 | 106 | 71 | 149 | 177 | 16 | 118 | 35 | 29 | 52 | 57 | 9 | 68 | 15 |
| **P2** | Mean | 93 | 87 | 329 | 444 | 711 | 767 | 98 | 166 | 226 | 90 | 79 | 91 | 25 | 92 | 39 |
|  | SD | 7 | 22 | 117 | 101 | 145 | 181 | 11 | 84 | 35 | 20 | 32 | 42 | 7 | 54 | 13 |
| **P3** | Mean | 97 | 80 | 359 | 503 | 743 | 845 | 97 | 170 | 226 | 93 | 86 | 90 | 24 | 98 | 43 |
|  | SD | 9 | 25 | 85 | 166 | 130 | 162 | 12 | 80 | 21 | 14 | 24 | 53 | 5 | 62 | 10 |
| **P4** | Mean | 91 | 79 | 326 | 446 | 659 | 680 | 103 | 131 | 212 | 87 | 88 | 83 | 25 | 86 | 42 |
|  | SD | 14 | 22 | 82 | 130 | 59 | 207 | 23 | 61 | 11 | 19 | 31 | 50 | 2 | 61 | 13 |
| **K1** | Mean | 93 | 82 | 312 | 267 | 465 | 434 | 93 | 107 | 158 | 59 | 84 | 106 | 34 | 89 | 40 |
|  | SD | 13 | 32 | 105 | 77 | 27 | 88 | 11 | 49 | 17 | 21 | 45 | 37 | 11 | 44 | 14 |
| **K2** | Mean | 96 | 99 | 360 | 485 | 707 | 702 | 108 | 168 | 232 | 92 | 94 | 105 | 32 | 95 | 42 |
|  | SD | 7 | 27 | 122 | 72 | 109 | 155 | 21 | 87 | 34 | 31 | 46 | 47 | 10 | 60 | 16 |
| **K3** | Mean | 96 | 89 | 335 | 421 | 679 | 741 | 102 | 162 | 220 | 89 | 86 | 94 | 31 | 94 | 40 |
|  | SD | 11 | 26 | 113 | 57 | 146 | 207 | 19 | 91 | 28 | 26 | 43 | 43 | 13 | 61 | 16 |
| **K4** | Mean | 92 | 87 | 340 | 425 | 679 | 744 | 98 | 164 | 226 | 94 | 83 | 96 | 26 | 95 | 40 |
|  | SD | 5 | 28 | 96 | 112 | 95 | 146 | 16 | 91 | 24 | 20 | 43 | 42 | 6 | 56 | 15 |
| **K5** | Mean | 95 | 82 | 360 | 469 | 707 | 800 | 99 | 176 | 227 | 92 | 91 | 94 | 25 | 96 | 40 |
|  | SD | 18 | 23 | 85 | 150 | 118 | 152 | 14 | 93 | 33 | 15 | 37 | 57 | 4 | 70 | 11 |
| **K6** | Mean | 98 | 81 | 349 | 497 | 675 | 800 | 102 | 170 | 226 | 88 | 92 | 95 | 26 | 98 | 44 |
|  | SD | 11 | 20 | 93 | 182 | 103 | 95 | 19 | 68 | 29 | 16 | 34 | 47 | 4 | 55 | 11 |

**Table S2: Mean and standard deviation (SD) of receptor density (in fmol/mg protein) in each layer of the V1.**

|  |  | **AMPA** | **kainate** | **NMDA** | **GABA_A_** | **GABA_B_** | **GABA_A_/BZ** | **M_1_** | **M_2_** | **M_3_** | **α_4_ꞵ_2_** | **α_1_** | **α_2_** | **5-HT_1A_** | **5-HT_2_** | **D_1_** |
| --- | --- | --- | --- | --- | --- | --- | --- | --- | --- | --- | --- | --- | --- | --- | --- | --- |
| **I** | Mean | 346 | 583 | 1441 | 1651 | 2168 | 1910 | 857 | 270 | 861 | 20 | 344 | 354 | 86 | 331 | 81 |
|  | SD | 49 | 72 | 146 | 302 | 424 | 324 | 380 | 76 | 147 | 5 | 82 | 24 | 57 | 74 | 15 |
| **II** | Mean | 416 | 646 | 1680 | 1958 | 2322 | 2260 | 1067 | 339 | 1018 | 23 | 362 | 420 | 74 | 370 | 83 |
|  | SD | 37 | 87 | 160 | 315 | 449 | 400 | 516 | 72 | 195 | 5 | 77 | 17 | 40 | 82 | 16 |
| **III** | Mean | 360 | 457 | 1735 | 2226 | 2197 | 2482 | 1153 | 424 | 1037 | 22 | 274 | 601 | 46 | 417 | 76 |
|  | SD | 59 | 51 | 145 | 359 | 487 | 414 | 559 | 82 | 218 | 3 | 67 | 23 | 19 | 104 | 15 |
| **IVA** | Mean | 287 | 394 | 1545 | 2164 | 1869 | 2446 | 1041 | 381 | 903 | 21 | 242 | 638 | 39 | 446 | 73 |
|  | SD | 56 | 40 | 140 | 377 | 449 | 462 | 536 | 87 | 239 | 3 | 63 | 46 | 18 | 115 | 13 |
| **IVB** | Mean | 245 | 313 | 1345 | 1903 | 1539 | 2188 | 835 | 352 | 727 | 20 | 196 | 436 | 46 | 456 | 70 |
|  | SD | 38 | 59 | 118 | 350 | 374 | 344 | 451 | 71 | 184 | 3 | 48 | 29 | 20 | 95 | 16 |
| **IVCα** | Mean | 222 | 305 | 1395 | 2043 | 1549 | 2316 | 768 | 427 | 701 | 29 | 195 | 440 | 55 | 519 | 75 |
|  | SD | 21 | 73 | 139 | 335 | 438 | 448 | 383 | 114 | 111 | 6 | 52 | 53 | 32 | 137 | 13 |
| **IVCß** | Mean | 249 | 529 | 1353 | 2072 | 1705 | 2435 | 777 | 440 | 750 | 29 | 234 | 534 | 61 | 515 | 81 |
|  | SD | 25 | 85 | 93 | 232 | 473 | 512 | 376 | 112 | 115 | 6 | 56 | 54 | 41 | 137 | 13 |
| **V** | Mean | 313 | 756 | 1149 | 1688 | 1686 | 2203 | 754 | 371 | 799 | 27 | 297 | 511 | 78 | 422 | 86 |
|  | SD | 42 | 91 | 120 | 282 | 417 | 432 | 340 | 91 | 137 | 6 | 66 | 39 | 40 | 91 | 14 |
| **VIa** | Mean | 352 | 783 | 992 | 1249 | 1444 | 1864 | 593 | 256 | 674 | 21 | 288 | 443 | 108 | 295 | 85 |
|  | SD | 60 | 96 | 69 | 243 | 238 | 393 | 262 | 85 | 94 | 3 | 50 | 59 | 46 | 88 | 17 |
| **VIb** | Mean | 235 | 461 | 653 | 776 | 910 | 1083 | 324 | 134 | 386 | 19 | 174 | 219 | 76 | 195 | 62 |
|  | SD | 73 | 61 | 132 | 299 | 147 | 250 | 169 | 70 | 79 | 4 | 52 | 47 | 36 | 80 | 15 |
